# *Leishmania (Viannia) braziliensis* infection in wild small mammals in ecotourism area of Brazil

**DOI:** 10.1371/journal.pone.0190315

**Published:** 2017-12-28

**Authors:** Gabriel Barbosa Tonelli, Aline Tanure, Felipe Dutra Rego, Gustavo Mayr de Lima Carvalho, Rodolfo Stumpp, Gabriela Ribeiro Ássimos, Aldenise Martins Campos, Ana Cristina Viana Mariano da Rocha Lima, Célia Maria Ferreira Gontijo, Gustavo Fontes Paz, José Dilermando Andrade Filho

**Affiliations:** 1 Grupo de Estudos em Leishmanioses, Instituto René Rachou, Fiocruz, Minas Gerais, Brasil; 2 Laboratório de Ecologia e Conservação, Departamento de Biologia Geral, Instituto de Ciências Biológicas, Universidade Federal de Minas Gerais, Minas Gerais, Brasil; Instituto Oswaldo Cruz, BRAZIL

## Abstract

Leishmaniases are parasitic diseases transmitted to mammalian hosts by sand fly vectors (Diptera: Psychodidae). Despite the increasing occurrence of visceral and cutaneous leishmaniasis cases in urban centers, their transmission still occur primarily in wild environments and may be associated with professional activities and recreation, such as ecotourism. The Reserva Particular do Patrimônio Natural Santuário do Caraça (RPPNSC) is one of the largest ecotourism attractions in the State of Minas Gerais, Brazil, and comprises an area of environmental preservation with 11,233 hectares presenting a transitional vegetation between Cerrado and Atlantic Forest. The present study describes the abundance of small mammals in RPPNSC, the isolation and identification of *Leishmania* in five wild animals. Small mammals were bimonthly trapped along 6 trails within the RPPNSC with 10 Tomahawk traps each. Two trails were located in peridomiciliary areas near tourist lodging facilities, and four trails were located at sites visited by tourists in forest areas. The most prevalent species were *Akodon cursor*, *Cerradomys subflavus* and Oligoryzomys nigripes. Six isolates of *Leishmania* were obtained from these animals and identified as *Leishmania braziliensis* through HSP70-PCR RFLP method. *Leishmania* spp. DNA was detected by kDNA-PCR method and isolated by biphasic culture. Studies point to some of the captured species as potential wild reservoirs of *Leishmania*, suggesting they may be involved in the transmission cycle in these wild environments.

## Introduction

Leishmaniasis is a complex of diseases caused by an intracellular protozoon, on vertebrate hosts, of the genus *Leishmania*. There are four generally accepted classifications of clinical leishmaniasis: cutaneous, diffuse cutaneous, mucocutaneous and visceral leishmaniasis. *Leishmania* infection is acquired through the bite of female sand flies of the genera *Phlebotomus* (Old World) and *Lutzomyia* (New World) [1]. In Brazil, the main species responsible for cutaneous leishmaniasis is *Leishmania (Viannia) braziliensis*, with approximately 26,000 new human cases reported every year [2], although it is difficult to obtain accurate numbers on the incidence of this disease.

The major reservoirs of this disease are small mammals, and many studies have been undertaken to determine the prevalence of infection by *Leishmania* species among small mammals of endemic and non-endemic areas [3, 4, 5, 6, 7]. Although the identification and incrimination of primary hosts of this species is complex, there are reports of infection in some wild and synanthropic mammals of different rodent genera in some States of Brazil including Minas Gerais, such as *Akodon* [8], *Rattus* [9], *Rhipidomys* [5, 10], *Nectomys* [11], *Necromys* (former *Bolomys*) [11, 12] and some marsupials [5, 13].

There is an increasing number of ecotourism areas nearby urban areas in the State of Minas Gerais, such as Ibitipoca State Park [14] and Rio Doce State Park [15], where important vectors of *Leishmania* were reported. In this context, the RPPNSC is inserted, one of the most visited parks in the state, surrounded by urban areas with autochthonous cases and lacking previous studies on possible wild hosts of *Leishmania*. In this study, we propose to investigate the *Leishmania* infection in small mammals caught in a natural protected area in southeastern Brazil which is surrounded by urban centers and constantly visited by tourists.

## Material and methods

The study was approved by the Ethics Committee for Research with Animals of the Oswaldo Cruz Foundation (CEUA/Fiocruz) under protocol number n° 14/16-3, and by the Brazilian Institute of Environment and Renewable Natural Resources (IBAMA) License n° 40191–2. All procedures involving experimental animals were conducted according to the guidelines of the Brazilian College for Experiments with Animals (Colégio Brasileiro de Experimentacão Animal/COBEA).

### Study area

**Fig 1:**
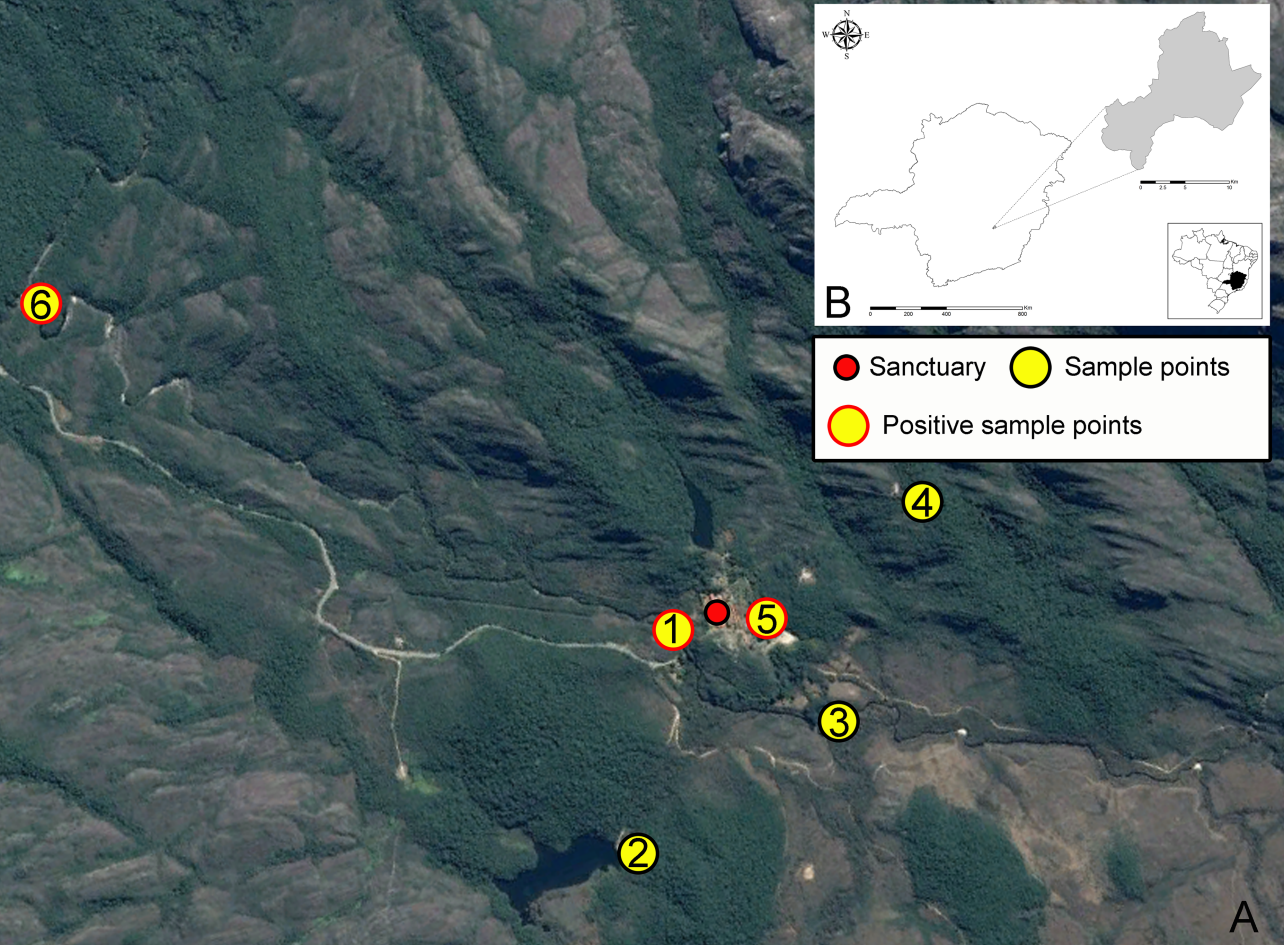
Sample sites and location site where isolation on biphasic medium culture and DNA detection by kDNA PCR of *Le*. *braziliensis* was performed from captured small mammals on the RPPN Santuário do Caraça. A—The red dot represents the location of the Sanctuary, yellow dots are the sample points and the red border in the yellow dot represents the location of isolation and detection of *Le*. *braziliensis*. B–Location of the RPPNSC in Brazil and Minas Gerais.

The present study was carried out in the “Reserva Particular do Patrimônio Natural (RPPN) Santuário do Caraça” (20°06’ S, 43° 27’ W), located between the cities of Santa Bárbara and Catas Altas in the Espinhaço mountain range in state of Minas Gerais, southeastern Brazil. This RPPN encompasses 11,233 hectares, of which 10,180 hectares are a protected area and 1,053 hectares of which are for sustainable management (Fig 1). Ecologically, the park comprises a transition zone between Atlantic Forest and Cerrado (Brazilian Savanna) with grasslands on mountain peaks. There are trails from the sanctuary (location of tourist lodging, dining, etc.) that lead to rivers, lakes, waterfalls, caves and peaks. This area remains one of the most popular tourist attractions in Brazil.

### Trapping of wild small mammals and sample collection

Small mammals were captured bimonthly, from July 2013 to July 2014, during four consecutive days per campaign. Ten Tomahawk® traps (30 x 17.5 x 15.5 cm) were placed at 10 m intervals along each of six transects (Fig 1). Traps were baited with a mixture of pineapple and emulsion of cod liver oil, which has been considered an effective attractant for wild small mammals [16, 5]. To perform parasitological and molecular tests for *Leishmania* detection by PCR-kDNA, all caught animals were anesthetized by a xylazine (10 mg / kg) and ketamine (200 mg / kg) followed by euthanasia by overdose of thiopental (1.25%) in concentration three times higher than that of the anesthetic plane and samples of spleen, liver, and skin (tail and ear) were collected. Identification of small mammal species followed specific literature [17, 18, 19]. In addition, they were compared to museum specimens. Collected specimens were deposited in the Mammal Collection of Universidade Federal de Minas Gerais (UFMG). Small-mammal nomenclature followed Paglia et al., [16].

### DNA extraction and PCR amplification

DNA extraction was performed using a Gentra Puregene QIAGEN (Germantown, MD, EUD) extraction kit following the manufacturer’s specifications.

PCR was conducted according to Degrave et al., [20] using the generic primers A: 5’ (C/G)(C/G)(G/C) CC(C/A) CTA T(T/A)T TAC ACC AAC CCC 3’ and B: 5’ GGG GAG GGG CGT TCT GCG AA 3’ in order to amplify a 120bp fragment of the conserved region within the minicircles of *Leishmania* kDNA [20]. Reaction mixtures were prepared in a final volume of 25uL containing 2uL of DNA template and 1× buffer solution (1.0mM Tris–HCl; 5.0mM KCl; 1.5mM MgCl2; pH 8.0), 200_M dNTPs, 10 pmol of each primer and 1.25U of Taq DNA polymerase (Invitrogen). The amplification conditions were as follows: 94°C for 4 min, followed by 30 cycles of denaturation at 94°C for 30 s, annealing at 60°C for 30 s and extension at 72°C for 30 s, with a final extension step at 72°C for 10min. Amplification were analysed on 2% polyacrylamide gels. All samples were tested for *irbp*-PCR as internal control for mammal DNA [21, 22] (S1 Appendix).

Amplified products were visualized in a 2% agarose gel stained with ethidium bromide, with a 100 bp DNA Step Ladder provided as a molecular-weight size-standard, and analyzed using the L-PIX EX (Loccus, Biotechnology, Cotia, SP) photo-documentation system. A panel of reference strains was used as a positive control, in all PCR procedures, which included *Leishmania amazonensis* (IFLA/BR/67/PH8), *Le*. *braziliensis* (MHOM/BR/75/M2903), *Le*. *infantum* (MHOM/BR/74/PP75) and *Le*. *guyanensis* (MHOM/BR/75/M4147).

### Culture and molecular characterization

Samples from spleen, liver, skin (tail and ear) were macerated in 1% phosphate-buffered saline (1 ml) with 50μL of Nistatin/100.000U/mL and penicillin/streptomycin antibiotic mix 100-200ųg/ml. After 24 hours macerated tissues were inoculated in NNN (Novy–MacNeal-Nicolle)/LIT (liver infusion tryptose) medium supplemented with 20% fetal bovine serum (FBS) and associated antibiotics (penicillin and streptomycin 100-200ųg/ml). All cultures were incubated at 25°C±1 about 50 days with periodic inoculation in new culture at 7-day intervals (a total of seven passages). The positive cultures were expanded to be cryopreserved (10% glycerol) and prepared for use in the different molecular techniques. The samples are deposited in the cryobank of the Laboratory of Leishmaniasis of the Centro de Pesquisas René Rachou. Negative cultures until the seventh week or contaminated by bacteria or fungi were discarded. DNA samples of positive cultures were subjected to PCR, targeting a fragment of the gene coding for heat shock proteins of 70 kilodaltons (*hsp70*) of *Leishmania* spp. using the primers HSP70 for 5’ GACGGTGCCTGCCTACTTCAA 3’ and HSP70rev for 5’ CCGCCCATGCTCTGGTACATC 3’, generating a 1300 bp fragment. Samples that had this specific band were subjected to digestion using the enzyme *Hae*III for analysis of restriction fragment length polymorphisms [23]. The restriction profiles were analyzed in 4% agarose and compared with standards of reference strains previously cited.

## Results

**Fig 2:**
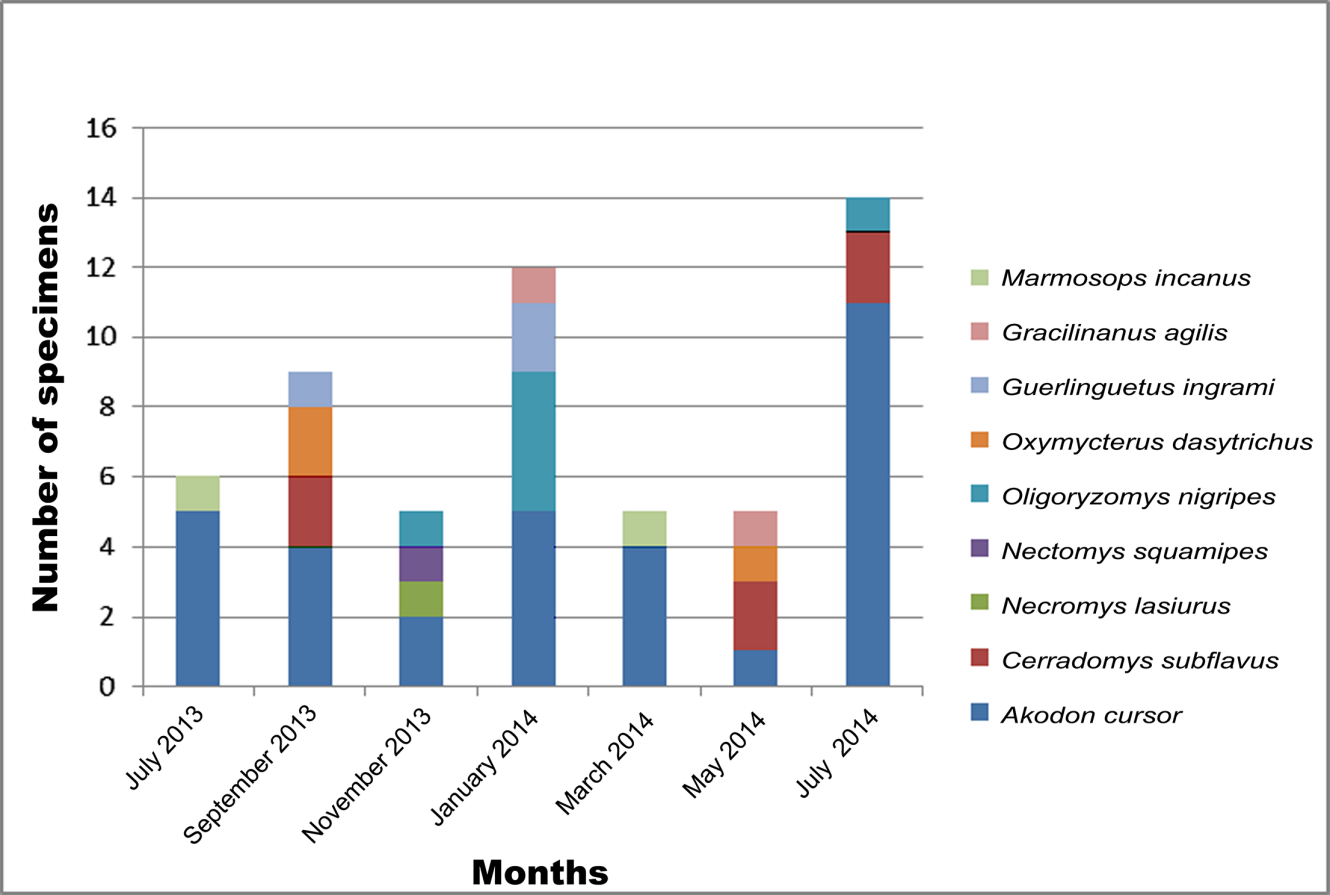
Frequency of trapped mammals in RPPN Santuário do Caraça between July 2013 and July 2014.

A total of 55 small mammals of six species of Cricetidae [*Akodon cursor* (Winge, 1887), *Cerradomys subflavus* (Wagner, 1842), *Necromys lasiurus* (Lund, 1841), *Nectomys squamipes* (Brants, 1827), *Oligoryzomys nigripes* (Olfers, 1818) and *Oxymycterus dasytrichus* (Schinz, 1821)], one species of Sciuridae [*Guerlinguetus ingrami* (Thomas, 1901)] and two species of Didelphidae [*Gracilinanus agilis* (Burmeister, 1854) and *Marmosops incanus* (Lund, 1841)] were captured during the study period (Fig 2). The most successful sampling points were the trail 1 (21,82%), trail 4 (20%) and trail 5 (20%) (Table 1). *Akodon cursor* (56.4%) was the most abundant species followed by *Cerradomys subflavus* (10.9%) and *Oligoryzomys nigripes* (10.9%) (Table 1).

**Table 1:** Captured small mammals by collection trails and the positivity on culture analysis.

Species	Trail 1	Trail 2	Trail 3	Trail 4	Trail 5	Trail 6	TOTAL (%)
	Captured animals (n of positive animals)						
Cricetidae							
*A*. *cursor*	9 (1^a^)	2	4	9	2 (1^b^)	5 (1^a^^b^)	31 (56,36)
*C*. *subflavus*	0	2	2	1	1 (1^a^)	0	6 (10,91)
*N*. *lasiurus*	0	0	1	0	0	0	1 (1,82)
*N*. *squamipes*	0	0	0	1	0	0	1 (1,82)
*O*. *nigripes*	1	3	0	0	2	0	6 (10,91)
*O*. *dasytrichus*	0	0	0	0	3 (1^b^)	0	3 (5,45)
Sciuridae							
*G*. *ingrami*	0	0	0	0	3	0	3 (5,45)
Didelphidae							
*G*. *agilis*	1	1	0	0	0	0	2 (3,64)
*M*. *incanus*	1	0	0	0	0	1	2 (3,64)
**TOTAL** **(%)**	**12 (21,82)**	**8** **(14,55)**	**7** **(12,73)**	**11 (20)**	**11 (20)**	**6 (10,90)**	**55** **(100)**

a—isolated parasite from tail skin

b—isolated parasite from liver

**Fig 3:**
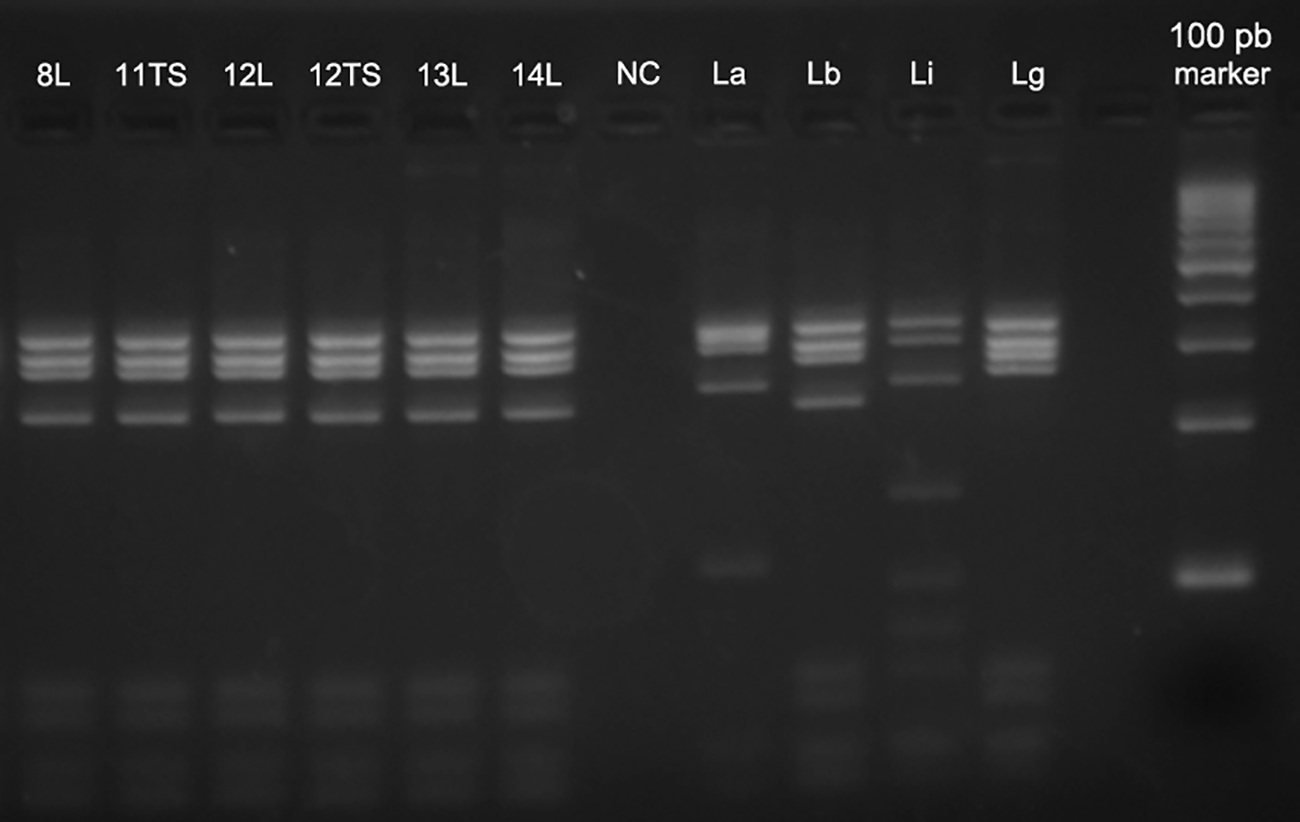
Electrophoresis 4% agarose gel of the RFLP HSP70 of positive DNA samples of small mammals collected in RPPNSC. MW = Molecular Weight, 8L, 11TS, 12L, 12TS, 13L and 14L = samples of liver (L) and tail skin (TS), NC = Negative Control, La, Lb, Lcand Lg = Positive Controls strains of *Leishmania amazonensis*, *Le*. *braziliensis*, *Le*. *infantum* and *Le*. *guyanensis* respectively.

Results found in culture recorded a total of 5 (9.1%) infected animals and six isolates. Isolates were obtained from liver and tail skin from three captured species (*Akodon cursor*, *Cerradomys subflavus*, *Oxymycterus dasytrichus*) and characterized by *hsp70* PCR-RFLP as *Leishmania (Viannia) braziliensis* (Fig 3). One culture was positive after 34 days and the others after 41 days. All positive culture samples were also positive by kDNA PCR (Table 2). The sample points where small mammals captured were positive on the culture and kDNA-PCR are marked on Fig 1.

**Table 2:** Species of small mammals captured in the RPPN Santuário do Caraça positive in kDNA PCR and culture by tissue analyzed.

Species	Liver		Spleen		Tail Skin	
	kDNA	Culture	kDNA	Culture	kDNA	Culture
*Cerradomys subflavus*	+	+				
*Akodon cursor*	+		+			+
*Akodon cursor*	+	+	+		+	+
*Akodon cursor*	+	+	+			
*Oxymycterus dasytrichus*	+	+				
**TOTAL**	**5**	**4**	**3**	**0**	**1**	**2**

## Discussion

Isolation of *L*. *braziliensis* from rodents has been described in different regions, for review see Roque & Jansen [24], but to the best of our knowledge, there are no prior reports of *L*. *braziliensis* isolated from *Akodon cursor*, *Cerradomys subflavus* and *Oxymycterus dasytrichus* in the southeastern region of Brazil. Here we report six isolations of *L*. *braziliensis* from these wild rodent species. Our results agree with the previous isolation of *L*. *braziliensis* from *Akodon arviculoides* [25] and *Nectomys squamipes* [26] in Brazil; and the cotton rat (*Sigmodon hispidus*) in Venezuela [27]. *L*. *braziliensis* has been also isolated from naturally infected *Necromys lasiurus* and *Rattus rattus* captured between 1996 and 2000 in the endemic cutaneous leishmaniasis region of Amaraji, Pernambuco State, northeast Brazil [3]. Additionally, in the same region an isolate was obtained from a spleen sample collected from a water rat (*Nectomys squamipes*). The spleen sample was initially inoculated in a hamster and then seeded on modified NNN medium. The isolate was characterized by monoclonal antibodies and multilocus enzyme electrophoresis as *L*. *braziliensis*, belonging to the zymodeme Z-74 the same one found in the isolates from ACL human cases [6].

In our study the animals showed *L*. *braziliensis* infection not only in the skin but also in the spleen and liver. It is important to note that this species of *Leishmania* has been previously reported to infect visceral organs [3, 6]. Our results also show that *Leishmania* infection in wild rodents follows a pattern different from that observed in human, infecting other tissues than the skin. This may change the point of view of the discussions about the dermotropic behavior of *L*. *braziliensis* as previously reported by Roque *et al*. [28].

Our results showed the same sensitivity for detection of *Leishmania* sp. in culture medium and molecular methods, contrary to literature data [3, 5]. Analysis of infection by *Leishmania* sp. in wild hosts can be made by tissue imprints on microscopy slides with results given in short time; however, sensitivity is highly variable (20%–70%) and requires experience for a praiseworthy diagnosis [29]. Culture in biphasic medium is another method to detect infection by parasites in hosts but it’s a laborious method and quite susceptible to contamination by fungi and bacteria, besides being a difficult methodology due to the parasite burden. The sensitivity of these methods, however, decreases as the number of parasites decreases, so PCR has become one of the most used method for detect infection by *Leishmania* spp. in small mammals, thus may be very useful in understanding the epidemiology of leishmaniasis [30]. The demonstrated capacity of *hsp70* PCR-RFLP analysis to distinguish among closely related *Leishmania* species, along with the ability to detect parasites in clinical samples, indicates that this technique could replace multilocus enzyme electrophoresis (MLEE) as the gold standard for *Leishmania* species identification [31].

None of the animals captured in the present study had a clinical sign of tegumentary leishmaniasis. Previous study found none of the small mammals examined to present clinical signs suggestive of leishmaniasis (e.g., skin lesions). This fact strengthen the hypothesis that these small mammals are suitable reservoirs for *L*. *braziliensis* and also suggests that these parasites have adapted to several small mammal species, probably due to their long co-existence [6], but new laboratory infection experiments must be taken to confirm this hypothesis.

Studies investigating the natural hosts of *Leishmania* parasites are often complicated due to the inherent difficulties in working with species of wildlife and the costs associated with long-term fieldwork [31]. However, the involvement of rodents in the maintenance of transmission cycle of *L*. *braziliensis* has been overlooked by public health and researchers, and is a component of the epizootiology of this disease that needs critical examination to update prevention and control programs in areas both endemic and non-endemic for tegumentary leishmaniasis.

Isolations were obtained from mammals captured on trails 1, 5 and 6, two peridomiciliary areas and one wild environment as showed on Fig 1. In one of the places where *L*. *braziliensis* was isolated from *Akodon* cursor, this same parasite was also detected by molecular methods in phlebotomine sand flies species *Psychodopygus lloydi* [32]. The isolations from vertebrate reservoirs were obtained on the month of September 2013, while the detection of *Leishmania* DNA in sand flies was made in December 2013, a month with greater density of sand flies [32].

Ours results in southeastern Brazil reinforce the hypothesis that rodents are major reservoir of *L*. *braziliensis*, and that *Cerradomys subflavus*, *Oxymycterus dasytrichus* and *Akodon cursor*, the most abundant species of rodent captured in the RPPN Santuário do Caraça and also with the high prevalence, are inserted in enzootic cycles of *L*. *brazilien*sis in the study area.

Some preventive care as use of repellents and appropriate clothing when practicing ecotourism may be adopted by visitors to reduce contact with mosquitoes that may act as vectors through the Santuário do Caraça. A very useful preventive measure already employed by the Sanctuary is the use of traps for small mammals in the peridomestic regions, preventing the circulation of these animals in the lodging areas. Continued studies in RPPN Santuário do Caraça are important for monitoring the situation of infections in local small mammals since it is an area of global tourist attraction, and thus can serve as a migration route for infectious diseases to non-endemic areas.

## Supporting information

S1 Appendix Electrophoresis 2% agarose gel of the *irbp*-PCR for control of the DNA extraction of small mammals collected in RPPNSC. MW = Molecular Weight, 1–13 = samples tested, NC = Negative Control.
(TIF)
