# Supplementary figures and images for: Leishmania (Viannia) braziliensis infection in wild small mammals in ecotourism area of Brazil
Source: PLoS One. 2017 Dec 28;12(12):e0190315. doi: 10.1371/journal.pone.0190315 (PMC5746269; doi:10.1371/journal.pone.0190315)

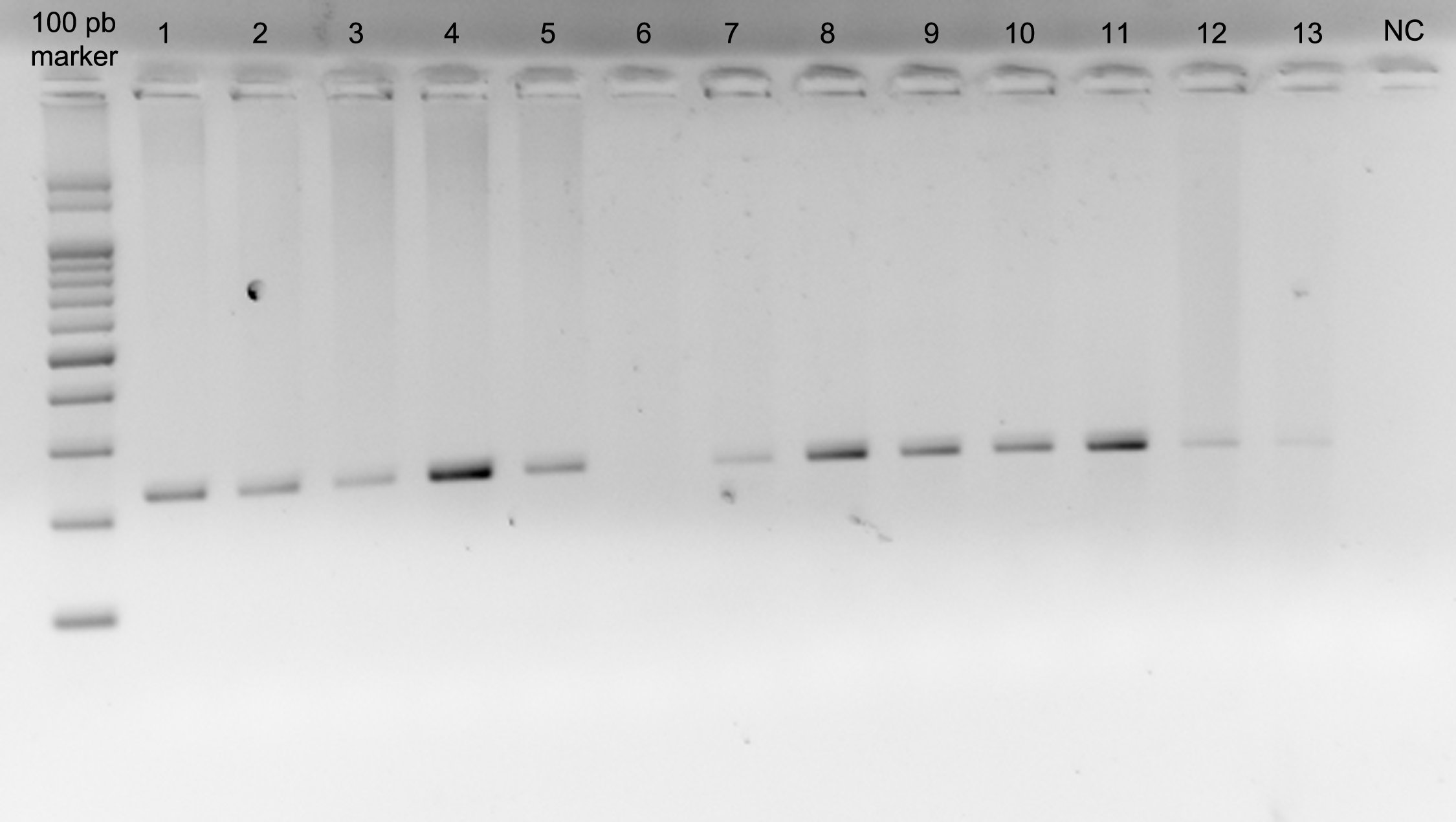

Supplement: S1 Appendix — MW = Molecular Weight, 1–13 = samples tested, NC = Negative Control. (TIF) [file pone.0190315.s001.tif]
